# Supplementary material for: Modular, On-Site Solutions with Lightweight Anomaly Detection for Sustainable Nutrient Management in Agriculture
Source: ACS ES T Eng. 2026 Feb 24;6(3):1089–105. doi: 10.1021/acsestengg.5c00635 (PMC12993859; doi:10.1021/acsestengg.5c00635)
Supplement: Supplementary file 1 [file ee5c00635_si_001.pdf]

Supporting Information

Modular, On-Site Solutions with Lightweight Anomaly Detection for Sustainable Nutrient Management in Agriculture

Cohen, Abigail R.[1]; Sun, Yuming[1]; Qin, Zhihao[2]; Muriki, Harsh S.[3]; Xiao, Zihao[4]; Lee, Yeonju[5]; Housley, Matthew[6]; Sharkey, Andrew F.[1]; Ferrarezi, Rhuanito Soranz[6]; Li, Jing[5]; Gan, Lu[7]; and Chen, Yongsheng[1*]

[1] School of Civil and Environmental Engineering, Georgia Institute of Technology, North Avenue, Atlanta, GA, United States 30332

[2] School of Electrical and Computer Engineering, Georgia Institute of Technology, North Avenue, Atlanta, GA, United States 30332

[3] School of Interactive Computing, Georgia Institute of Technology, North Avenue, Atlanta, GA, United States 30332

[4] School of Computational Science and Engineering, Georgia Institute of Technology, North Avenue, Atlanta, GA, United States 30332

[5] School of Industrial and Systems Engineering, Georgia Institute of Technology, North Avenue, Atlanta, GA, United States 30332

[6] Department of Horticulture, University of Georgia, Athens, GA, United States 30602

[7] School of Aerospace Engineering, Georgia Institute of Technology, North Avenue, Atlanta, GA, United States 30332

[*] Corresponding Author, yongsheng.chen@ce.gatech.edu

CONTENTS

SI 1 Additional Experimental Information 3
SI 1.1 Grow Environment 3
SI 1.1.1 Fertilizer Nutrient Composition 4
SI 1.2 Tissue Analysis 4
SI 1.3 Water Analysis 4
SI 1.4 Imaging Setup 5
SI 1.5 Image Preprocessing 5
SI 1.6 Additional Details on Vegetation Index (VI) Calculation 7
SI 2 Additional Model Details 9
SI 2.1.1 Random Forest Feature Selection 9
SI 2.1.2 Additional ViT Preprocessing, & Data Augmentation 9
SI 2.1.3 Details of State Estimation Architectures 9
SI 3 Additional Results 11
SI 3.1 Plant Tissue Analysis Results 11
SI 3.2 Additional Model Results 12
SI 3.2.1 Important Features in RF 12
SI 3.2.2 Time-Series Estimated Response Variable Phenotypes from the RF Model 12
SI 3.3 Statistical Analysis Results 16

SI 1 ADDITIONAL EXPERIMENTAL INFORMATION

SI 1.1 Grow Environment

Rex lettuce (*Lactuca sativa*) seeds were germinated in 1-inch grow cubes for two weeks and then transplanted into net cups and placed in the 9 deepwater culture tanks on October 15, 2024. 72 plants were allotted to each tank, spaced 8-inch apart, for a total of 648 plants. The greenhouse environment was maintained across all treatments and tanks with a STEP-Up Control system (Wadsworth Control Systems, Arvada, CO, USA). pH was maintained between 5.5 and 6.5 using stock pH Up and pH Down (Advanced Nutrients, West Hollywood, CA, USA). KOH (35-50 %) and H_3PO_4 (60-90%) additions were consistent within treatment classes. Water levels were manually checked and maintained at roughly 290 L using known water volumes 2-3 times per week as needed.

Greenhouse environmental setpoints: 80% humidity, nighttime temperature of 68º F, with cooling initiated above 70ºF or heating below 67ºF. The daytime temperature was set to 76ºF, with cooling initiated above 79ºF or heating below 75ºF.

SI 1.1.1 Fertilizer Nutrient Composition

Stock solution was diluted using a water-powered dose injector (D14MZ2 - 14 GPM, Dosatron International S.A.S., Tresses, France) at 1:100, 1:200, and 1:400 for T1, T2, and T3, respectively.

Table SI 1: Fertilizer nutrient composition for three treatment groups

	**Stock Solution**	**Treatment Solutions***		
**Fertilizer Salts**	**g/18.9L**	**T1 (100%) mg/L**	**T2 (50%) mg/L**	**T3 (25%) mg/L**
Calcium Nitrate	900	476.19	238.10	119.05
Ammonium Nitrate	70	37.04	18.52	9.26
Monopotassium Phosphate (MKP)	170	89.95	44.97	22.49
Magnesium Sulfate	200	105.82	52.91	26.46
Potassium Nitrate	500	264.55	132.28	66.14
Boric Acid	1.73	0.91	0.46	0.23
Copper Sulfate	0.15	0.08	0.04	0.02
Fe-EDDHA	62	32.80	16.40	8.20
Manganese Sulfate	1.5	0.79	0.40	0.20
Zinc Sulfate	0.69	0.37	0.18	0.09
Ammonium Molybdate	0.07	0.04	0.02	0.01
Monoammonium Phosphate (MAP)	75	39.68	19.84	9.92
Magnesium Nitrate	500	264.55	132.28	66.14

**Concentrations prior to pH adjustment*

pH was controlled as needed using stock pH Up and pH Down (Advanced Nutrients, West Hollywood, CA). KOH (%) and H3PO4 (%) additions were consistent within treatment groups.

SI 1.2 Tissue Analysis

Total nitrogen (%) was obtained using a LECO-Nitrogen Gas analyzer (Bernie B. Bernard, Heather Bernard, and James M. Brooks TDI-Brooks International/B&B Laboratories Inc. College Station, Texas 77845). Remaining elements (P, K, Ca, Mg, S) were quantified using inductively coupled argon plasma (ICP) using wet digestion and a DigiBloc 3000 (SPC Science), DigiTubes, and watch glasses.

SI 1.3 Water Analysis

Ammonia-N was quantified using flow injection analysis (FIA) using a FIALYZER-1000 and Cetac ASX-560 auto-sampler. Their FIA method used a reagent system comprised of sodium hydroxide-DTPA-K_2SO_4 carrier solution, hypochlorite solution, and salicylate-nitroferricyanide solution. Calibration

standards of 0, 0.5, 1.0, 5.0, and 10.0 ppm NH_4-N. Nitrate-N was quantified using cadmium reduction spectrophotometry (Water Analysis Handbook Method 8039) with a detection range of 0.3-30 ppm NO_3-N and a dilution protocol for concentrations above 30 ppm as needed up to 150 ppm with correction factor. Phosphorus, potassium, calcium, magnesium, sulphate, boron, zing, manganese, iron, and copper were quantified using ICP-AES following EPA Method 200.7.

SI 1.4 Imaging Setup

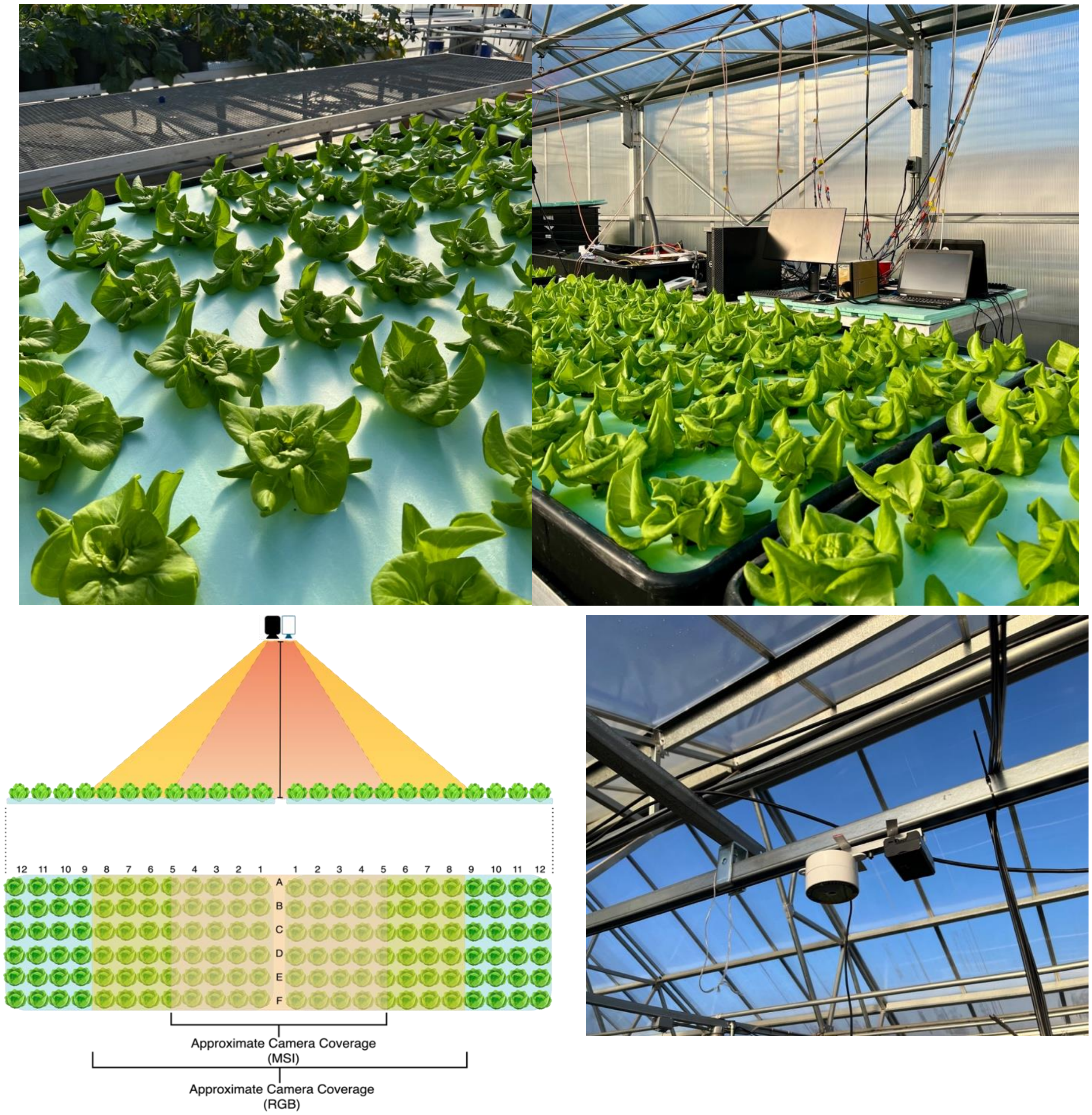

Figure SI 1: Approximate coverage for multispectral imaging (MSI) cameras, for 4 of 5 cameras (top left); a photo of the imaging setup (top right); coverage for T13 (bottom left) tank was larger than T11, T12, T21-T23, and T31-T33 (example, T32 and T31, pictured in bottom right).

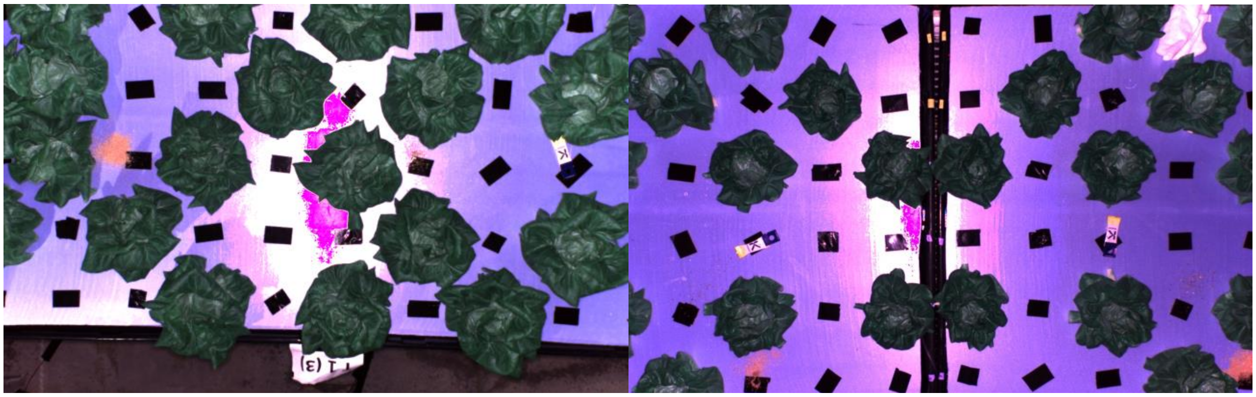

Figure SI 2: Example overhead MSI images for T13 (left) and T11 and T12 (right).

SI 1.5 Image Preprocessing

The preprocessing pipeline for the Multispectral Imaging (MSI) data was completed using the following steps:

1. Convert MSI to RGB for SAM input.
2. Run SAM Segmentation using the RGB image as described above.
3. Apply the mask to original MSI to isolate plant-level MSI data.
4. Output: .npy files containing masked MSI data per plant.

A diagram detailing these steps can be found in Figure SI 3.

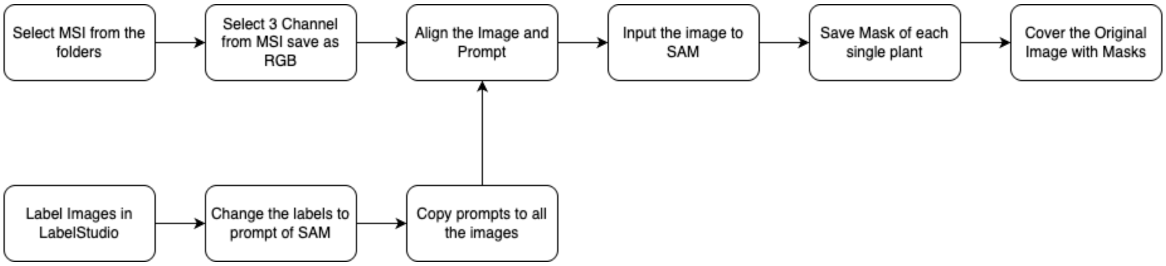

Figure SI 3: Summary of the preprocessing pipeline using SAM for masking and segmentation.

In addition to the automated segmentation pipeline, denoising was also required for some images, especially in the event of occlusion when plants developed to the point where their leaves overlapped. To ensure accuracy, manual post-processing for correction was required in some cases.

SI 1.6 Additional Details on Vegetation Index (VI) Calculation

The VI calculation pipeline was implemented as a four-stage workflow. First, the raw MSIs were loaded by a reader. Next, each loaded data cube was passed to the Channel Separator and split the array into individual wavelength channels. For MS index calculations, only six principal bands (blue, green, red, red-edge, near-infrared, and shortwave-infrared) were used for downstream indices. Following preparation, a the VIExtractor class was used to extract the VIs. A 'valid-pixel' mask was first created by thresholding the sum of spectral bands (e.g., R + G + B + NIR + RE) to exclude background regions. Pixel-count metrics (valid vs. background) were recorded, and simple per-band statistics (mean, median, standard deviation, minimum, maximum, and range) were computed over only those valid pixels. Subsequently, both multispectral indices and RGB-based indices were calculated (Table SI 2). Finally, the DataSaver class integrated image metadata and index results into a single, flat table. By parsing the parent-folder and filename via regular expressions, it extracts experimental identifiers (like tank and plant IDs) and precise timestamps. These metadata was then written to a CSV file in one batch. The end-to-end design—from raw images to standardized output—ensured that every step the vegetation-index pipeline is transparent, fully reproducible, and readily adaptable to new indices or image sensors. A total of 106 VI-based features were calculated and used for subsequent analysis.

Table SI 2: RGB and MSI Indices used for feature derivation in this study.

Index	Abbrv	Formula	Reference
**RGB-Vegetation Indices**			
Excess Green	ExG	$\boldsymbol{ExG = 2\,G_f - R_f - B_f}$	[1]
Excess Green minus Excess Red	ExGR	$\boldsymbol{ExGR = ExG - (1.4\,R_f - G_f)}$	[2]
Normalized Green-Red Difference Index	NGRDI	$\boldsymbol{NGRDI = (G_f - R_f)/(G_f + R_f)}$	[3]
Normalized Blue-Red Difference Index	NGBDI	$\boldsymbol{NGBDI = (G_f - B_f)/(G_f + B_f)}$	[4]
Red-Green Ratio Index	RGRI	$\boldsymbol{RGRI = R_f/G_f}$	[5]
Green-Blue Ratio Index	GBRI	$\boldsymbol{GBRI = B_f/G_f}$	[6]
Color Index of Vegetation Extraction	CIVE	$\boldsymbol{CIVE = 0.441R_f - 0.811G_f + 0.385B_f + 18.787}$	[7]
Red, Green Blue Vegetation Index	RGBVI	$\boldsymbol{RGBVI = ({G_f}^2 - (B_f * R_f))/({G_f}^2 + (B_f * R_f))}$	[8]
Modified Green Red Vegetation Index	MGRVI	$\boldsymbol{MGRVI = ({G_f}^2 - {R_f}^2)/({G_f}^2 + {R_f}^2)}$	[8]
**Multispectral-Vegetation Indices**			
Normalized Difference Vegetation Index	NDVI	$\boldsymbol{NDVI = (NIR_f - R_f)/(NIR_f + R_f)}$	[9]
Ratio Vegetation Index	RVI	$\boldsymbol{RVI = NIR_f/R_f}$	[3]
Green Normalized Difference Vegetation Index	GNDVI	$\boldsymbol{GNDVI = (NIR_f - G_f)/(NIR_f + G_f)}$	[10]
Green and Red Ratio Vegetation Index	GRVI	$\boldsymbol{GRVI = NIR_f/G_f}$	[11]
Normalized Difference Water Index	NDWI	$\boldsymbol{NDWI = (NIR_f - SWIR_f)/(NIR_f + SWIR_f)}$	[12]
Normalized Difference Red Edge	NDRE	$\boldsymbol{NDRE = (NIR_f - RE)/(NIR_f + RE)}$	[13,14]

SI 2 ADDITIONAL MODEL DETAILS

SI 2.1.1 Random Forest Feature Selection

First, recursive feature elimination (RFE) was applied to retain only the top 20% of features based on their contribution to predictive performance. Next, features with importance scores below 0.01 were removed to eliminate variables with minimal influence. Finally, correlation pruning reduced redundancy among remaining features. Specifically, for each pair of features with a Pearson correlation coefficient greater than 0.95, the feature with lower importance was dropped only if its importance was below 0.05 and if its removal resulted in an increase in validation R^2 of at least 0.002 or a decrease in validation RMSE by at least 1%. Hyperparameter tuning was conducted independently within each fold using a random search approach. A total of 50 hyperparameter combinations were randomly sampled from a predefined grid, and the combination that yielded the highest validation R^2 was selected for that fold.

SI 2.1.2 Additional ViT Preprocessing, & Data Augmentation

Upon initialization, data augmentation was applied using the Numpy.transforms library to increase generalizability and reduce overfitting. Transforms applied to the training set with probability p (p = 0.5), included: (1) random horizontal flip; (2) random vertical flip; (3) random rotations of 0º, 90º, 180º, 270º degrees; and (4) numpy to tensor. To tune the model, the transforms applied to the validation set included only tensor conversion. No transformations were applied to test data.

SI 2.1.3 Details of State Estimation Architectures

Table SI 3: Random Forest hyperparameter values.

Hyperparameters	N	P	K	Ca	Mg	S	FW	DM
estimators (n)	900	1300	900	900	900	1300	900	900
max depth	14	12	14	14	14	12	14	14

All response variable models used the following:
Min samples split: 2
Min samples leaf: 1
Max Features: sqrt
Bootstrap: FALSE
Criterion: Squared Error

Table SI 4: Architecture details for the ViT model.

Layer (type:depth-idx)	Input Shape	Output Shape	Param #
ViTModel	[32, 10, 224, 224]	[32, 8]	--
├─ChannelProjector: 1-1	[32, 10, 224, 224]	[32, 10, 224, 224]	110
├─ViT: 1-2	[32, 10, 224, 224]	[32, 256]	50,688
│ └─Sequential: 2-1	[32, 10, 224, 224]	[32, 196, 256]	--
│ │ └─Rearrange: 3-1	[32, 10, 224, 224]	[32, 196, 2560]	--
│ │ └─LayerNorm: 3-2	[32, 196, 2560]	[32, 196, 2560]	5,120
│ │ └─Linear: 3-3	[32, 196, 2560]	[32, 196, 256]	655,616
│ │ └─LayerNorm: 3-4	[32, 196, 256]	[32, 196, 256]	512
│ └─Dropout: 2-2	[32, 197, 256]	[32, 197, 256]	--
│ └─Transformer: 2-3	[32, 197, 256]	[32, 197, 256]	--
│ │ └─ModuleList: 3-5	--	--	--
│ │ │ └─ModuleList: 4-1	--	--	1,575,424
│ │ │ └─ModuleList: 4-2	--	--	1,575,424
│ │ │ └─ModuleList: 4-3	--	--	1,575,424
│ │ │ └─ModuleList: 4-4	--	--	1,575,424
│ │ │ └─ModuleList: 4-5	--	--	1,575,424
│ │ │ └─ModuleList: 4-6	--	--	1,575,424
│ │ └─LayerNorm: 3-6	[32, 197, 256]	[32, 197, 256]	512
│ └─Identity: 2-4	[32, 256]	[32, 256]	--
│ └─Linear: 2-5	[32, 256]	[32, 256]	65,792
├─Sequential: 1-3	[32, 256]	[32, 128]	--
│ └─Linear: 2-6	[32, 256]	[32, 128]	32,896
│ └─ReLU: 2-7	[32, 128]	[32, 128]	--
├─Linear: 1-4	[32, 128]	[32, 8]	1,032

```
Total params: 10,264,822
Trainable params: 10,264,822
Non-trainable params: 0
Total mult-adds (M): 326.85

Input size (MB): 64.23
Forward/backward pass size (MB): 1716.42
Params size (MB): 40.86
Estimated Total Size (MB): 1821.50
```

The MSI-ViT model also used the following settings:

- Batch size:32
- Input channels: 10
- Learning rate: Variable, initialized at 0.00001
- Number of epochs:100
- Number of outputs:8
- Number of workers:1
- Output channels:10
- MLP dimensions: 1024
- Dimension of patch embedding: 256
- Number of transformer encoder layers: 6 (depth in the model)
- Number of attention heads and the output dimension of MLP for each encoder layer: Attention heads = 16; MLP Hidden Dimensions = 1024, Model Embedding Dimensions = 256
- Dimension of the fully connected layer (FCL):
    - **Input to FCL: 256 (from ViT backbone)**
    - Hidden FCL: 256 → 128
    - Output FCL: 128 → number of outputs (8 response variables)

SI 3 ADDITIONAL RESULTS

SI 3.1 Plant Tissue Analysis Results

Plant tissue analysis was completed in November and December of 2024. Results indicate that early growth is not dependent on substrate concentration, but by 14 DAT there is a clear divergence in fresh weight of the T3 treatment class (25%) from the other two groups, followed by a divergence in fresh weight of T2 (50%) from the control, T1 (100%). This can be seen in the box-and-whisker plots Figure SI 4.

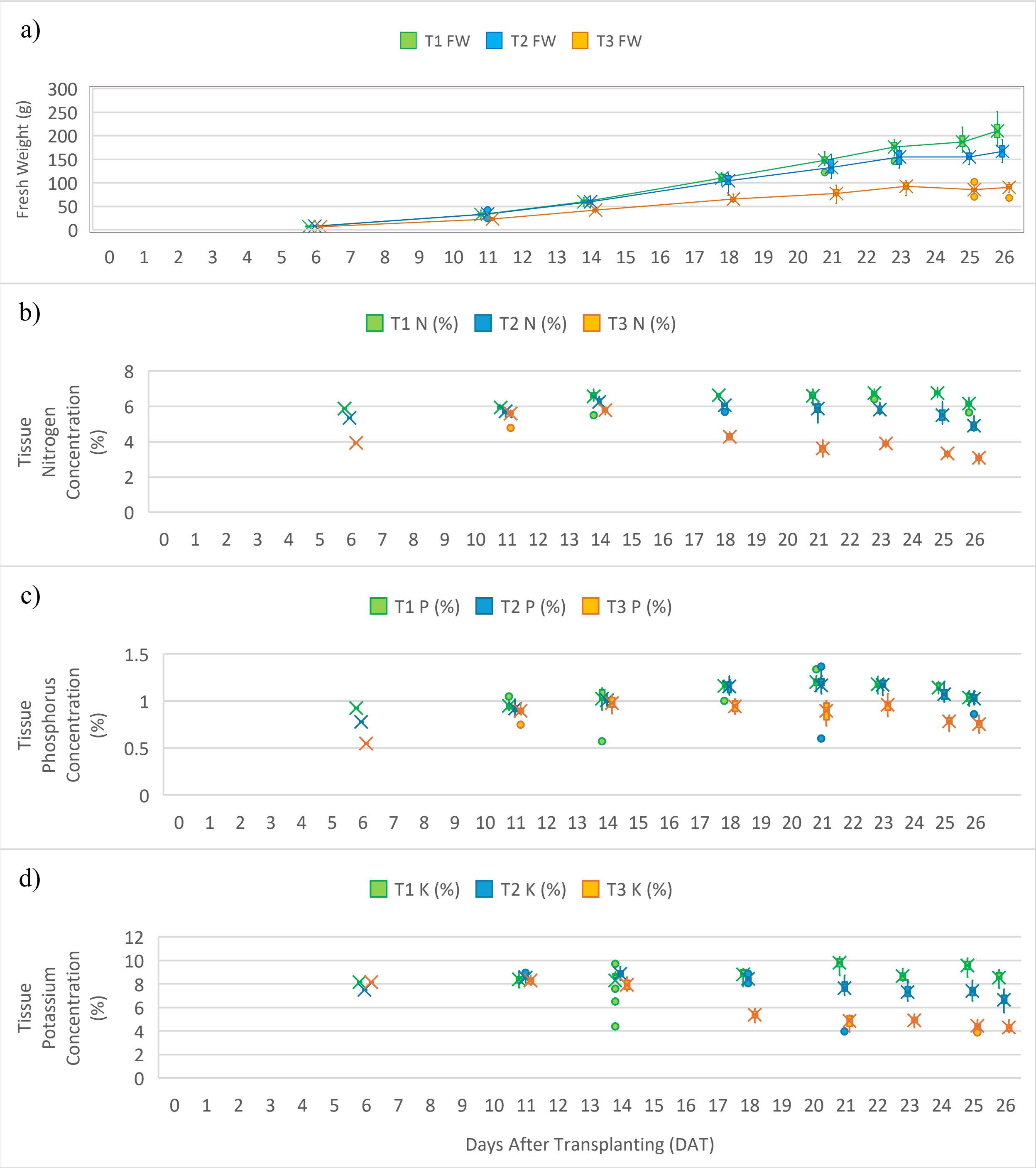

Figure SI 4: Box-and-whisker plots for fresh weight (a), tissue nitrogen (N) (b), tissue phosphorus (P) (c), and tissue potassium (K) (d).

Figure SI 4 shows box-and-whisker plots of tissue analysis of N, P, and K in the three treatments. Each show much greater deviation in the T3 treatment class from T1 and T2 than in T2 from T1. These profiles show a non-linear relationship between concentration and uptake and the subsequent growth of tissue, with a plateau in treatment impact with increasing concentration. This supports evidence that plant uptake

and growth follow logistic growth, similar to Michaelis-Menten or Monod-type kinetics, with a saturation concentration above which uptake and growth have little impact.

SI 3.2 Additional Model Results

SI 3.2.1 Important Features in RF

Using the RFE pipeline outlined in 2.3.1, important features were determined for each of the 8 response variable phenotypes, which are pictured using SHAP for N, P, K, and Ca (Figure SI 5).

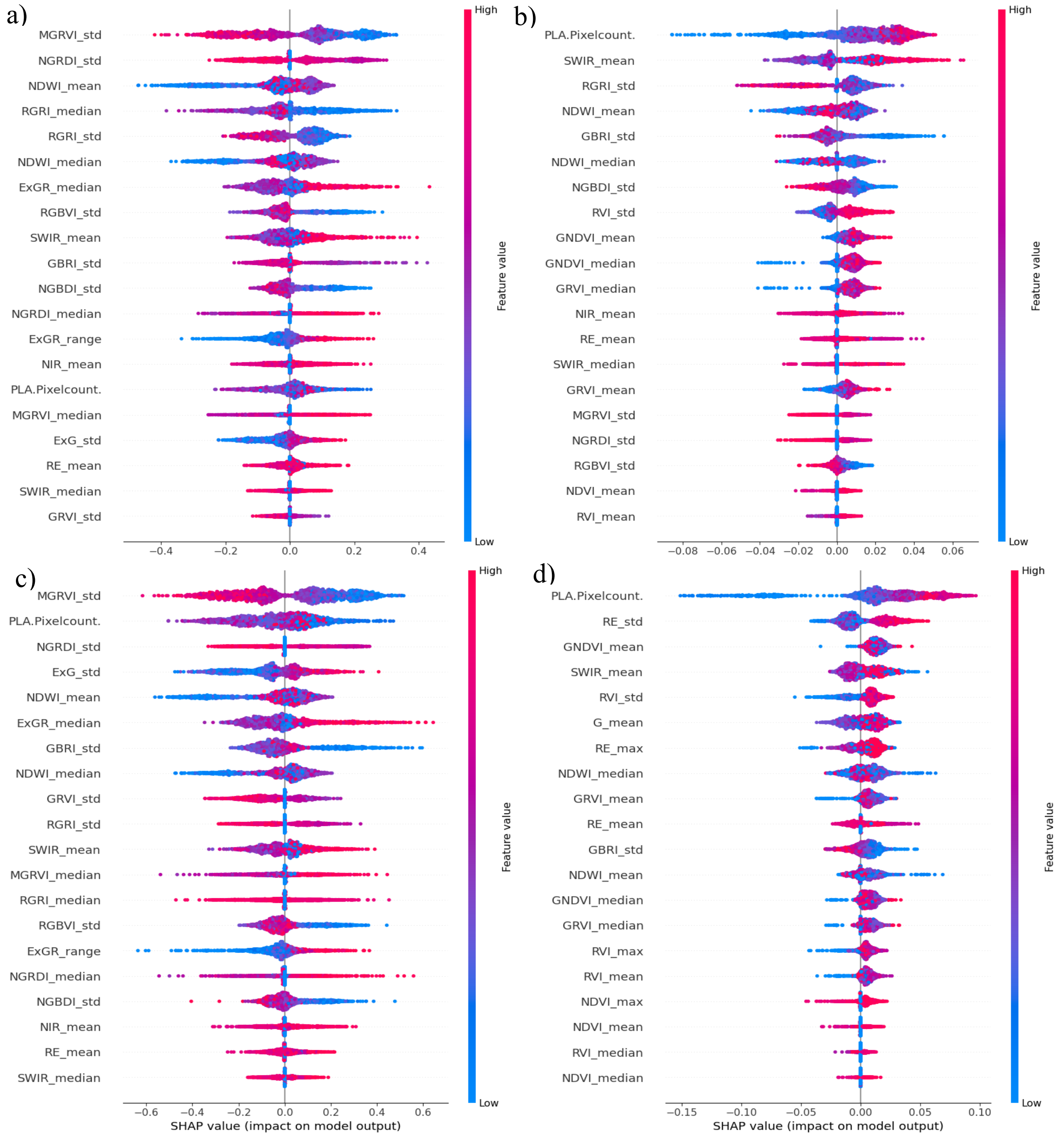

Figure SI 5: SHAP analysis of important features for RF from RFE for nitrogen (a), phosphorus (b), potassium (c) and calcium (d). Feature importance value, from low to high, is indicated in cooler to warmer color, while SHAP value from negative to positive intensity is found on the x-axis.

The most important feature for N and K estimation was MGRVI_std, with a strong negative correlation, indicating that the distribution of this VI over the foliar surface is more important for estimating N and K than the value itself. This means foliar surface MGRVI values of plants with lower tissue concentrations

display more variation. Meanwhile, projected leaf area (PLA) was most important for P and Ca, meaning the overall size of the sample is the strongest indicator for Ca and P concentrations.

SI 3.2.2 Time-Series Estimated Response Variable Phenotypes from the RF Model

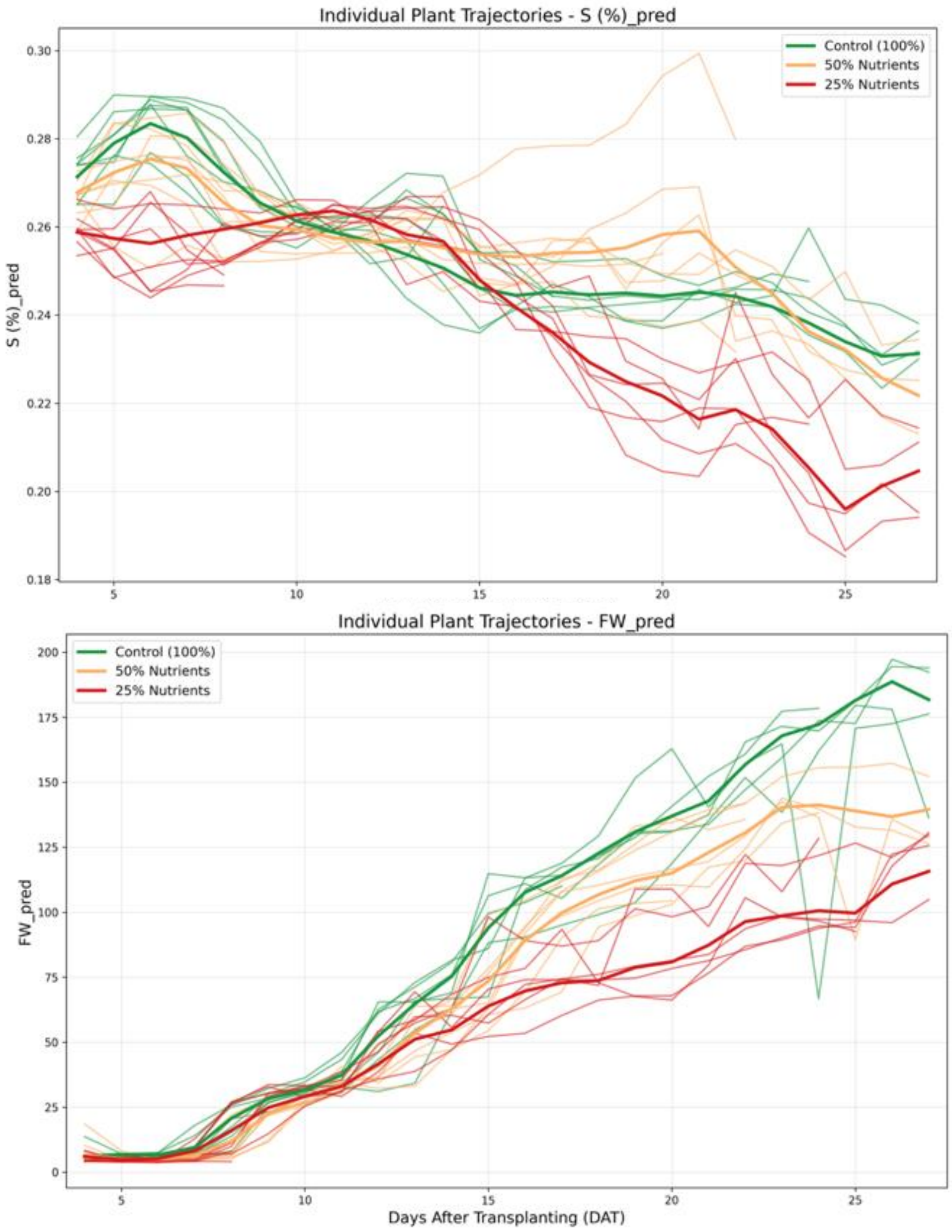

Figure SI 6: Selection of 8 predicted trajectories estimated by the random forest model for all 7 response variables for T1 (green), T2 (yellow), and T3 (red) treatments for S (top) and FW (bottom).

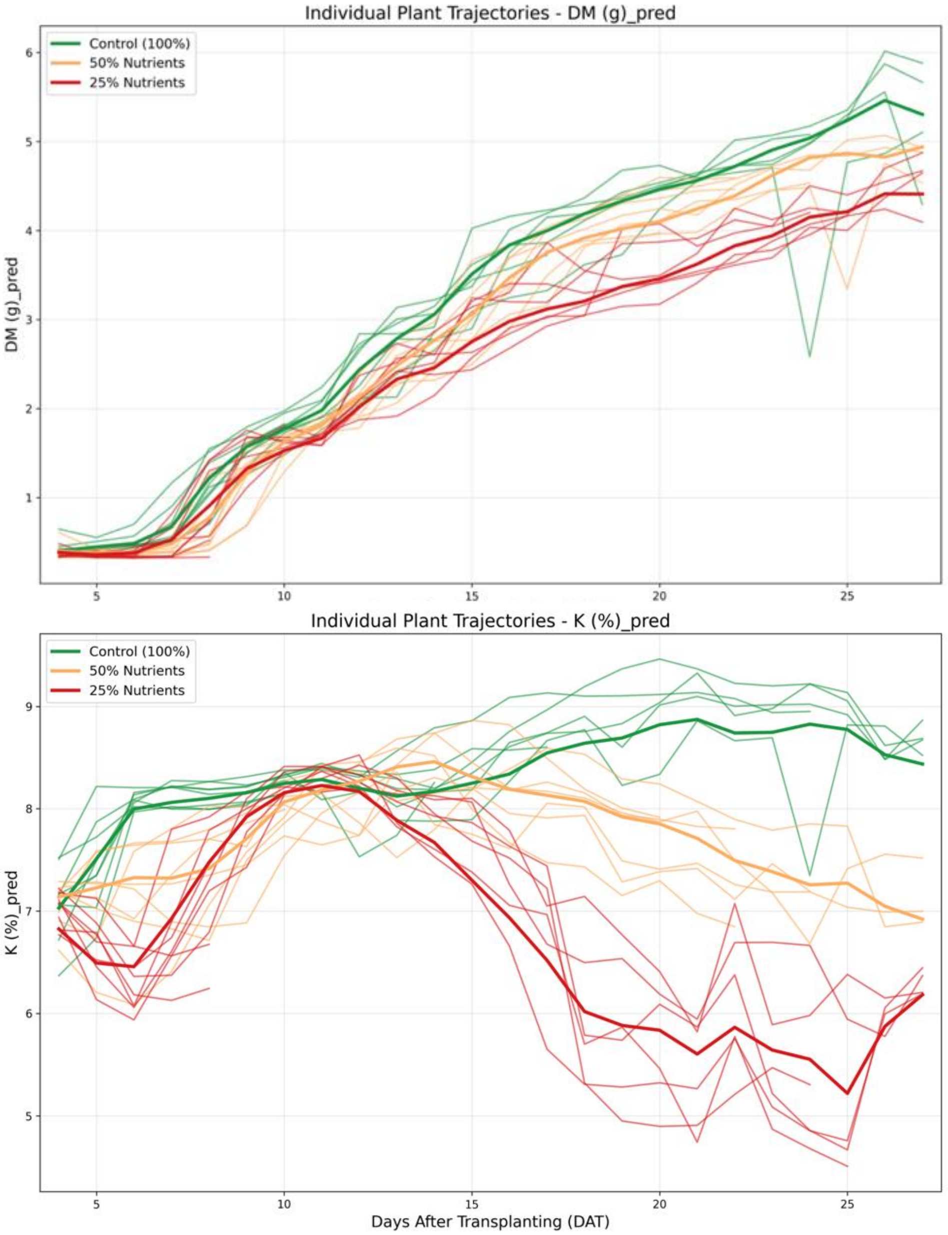

Figure SI 7: Selection of 8 predicted trajectories estimated by the random forest model for all 7 response variables for T1 (green), T2 (yellow), and T3 (red) treatments for DM (top) and K (bottom).

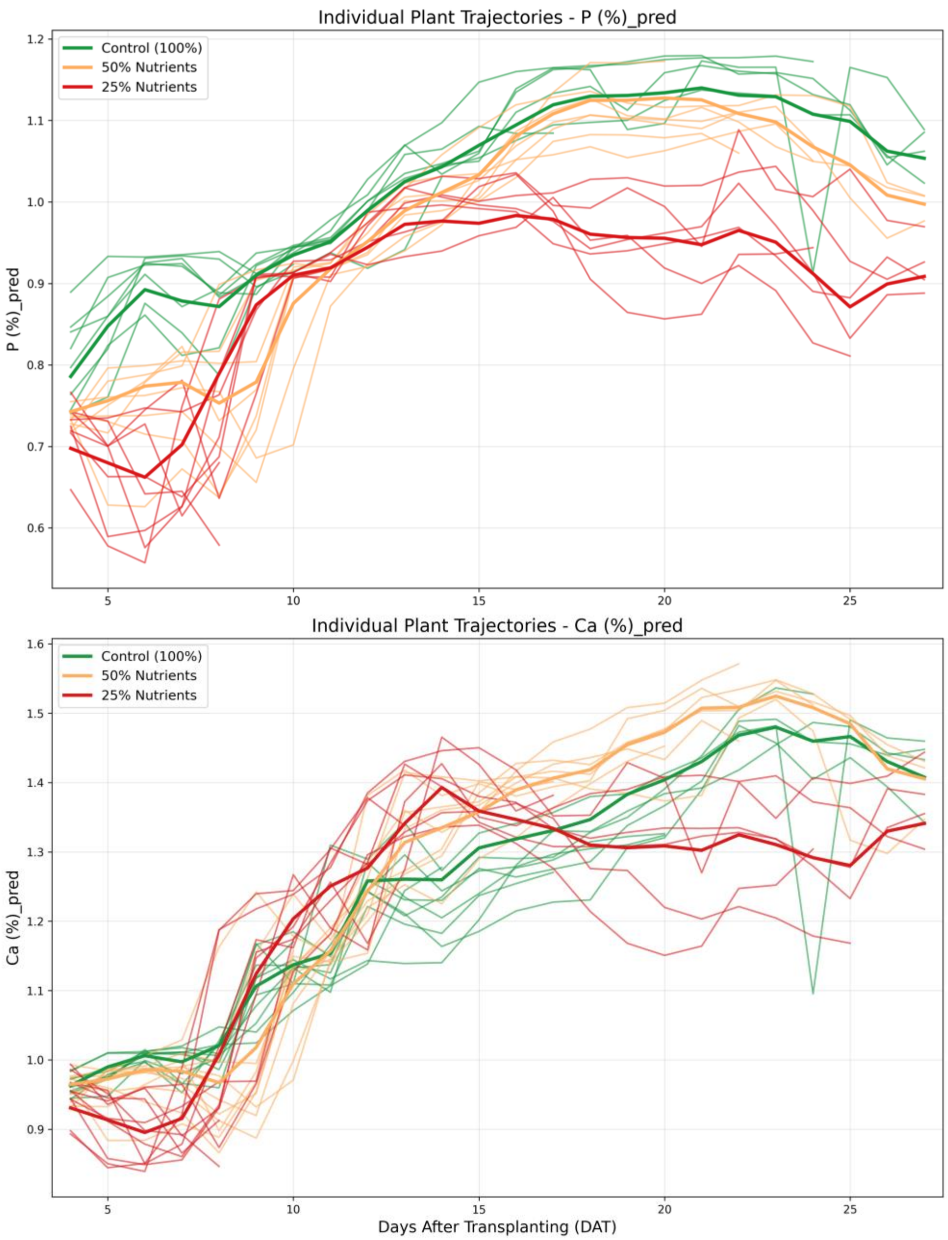

Figure SI 8: Selection of 8 predicted trajectories estimated by the random forest model for all 7 response variables for T1 (green), T2 (yellow), and T3 (red) treatments for P (top) and Ca (bottom).

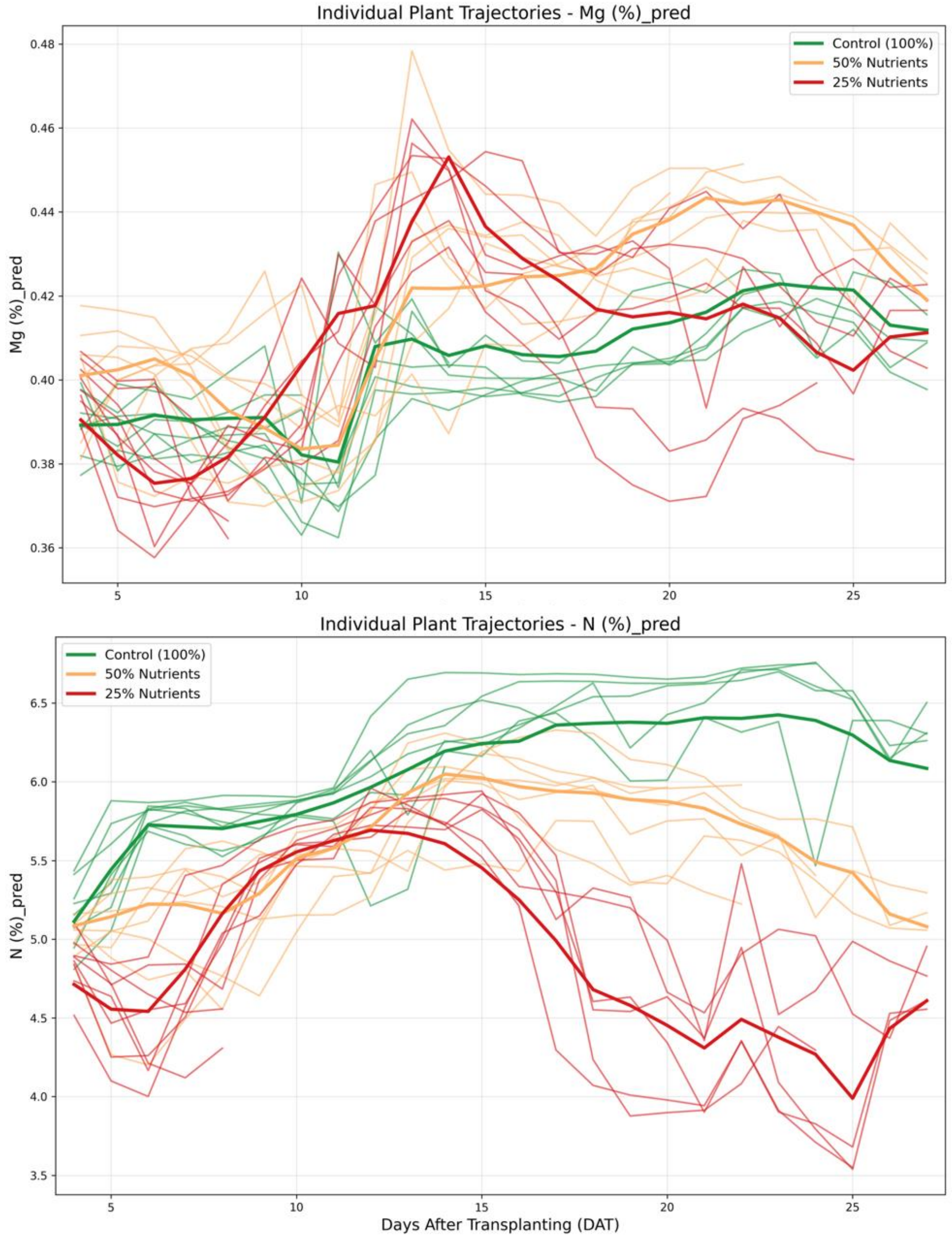

Figure SI 9: Selection of 8 predicted trajectories estimated by the random forest model for all 7 response variables for T1 (green), T2 (yellow), and T3 (red) treatments for Mg (top) and N (bottom).

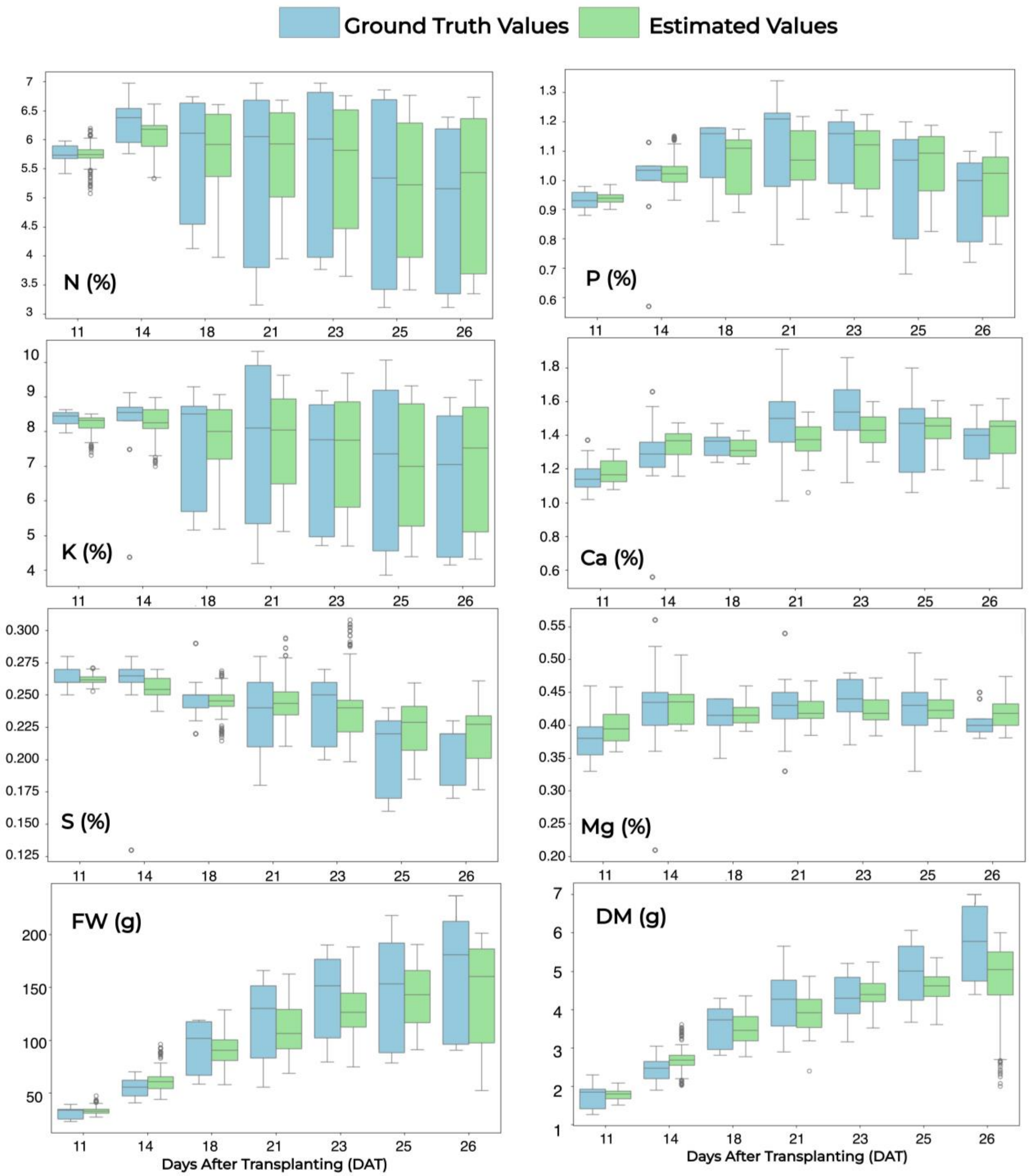

Figure SI 10: Estimated response variables over time vs. the ground truth analytical values for responses on each of the 7 sampling occasions for all 8 response variables.

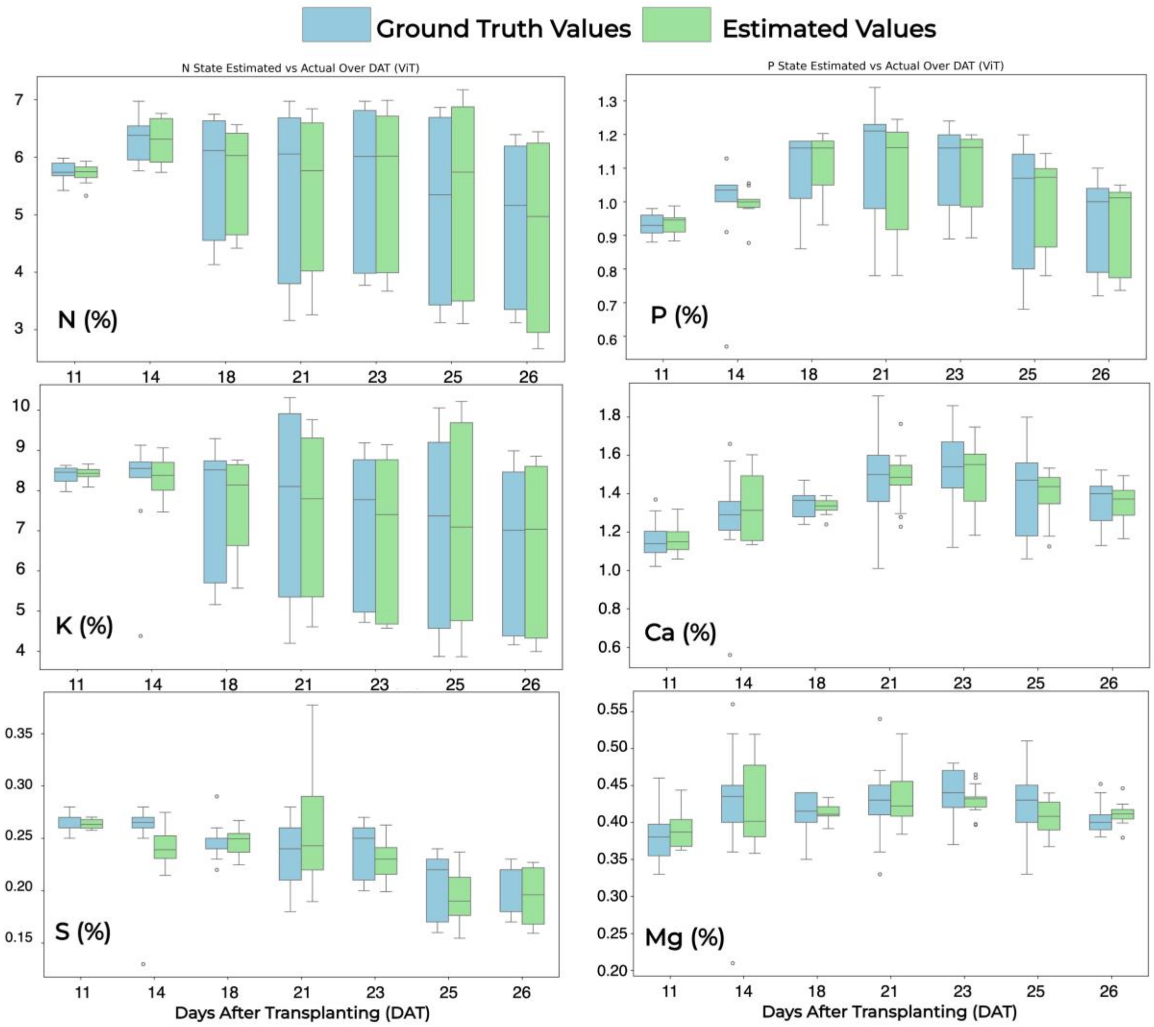

Figure SI 11: MSI-ViT accuracy versus biological variability for each tissue nutrient at each sampling occasion.

SI 3.3 Statistical Analysis Results

Tissue Analysis Data Variance for GT and Estimated data. Visualizations of treatment effects significance over time can be found in Figure SI 12. These results for N, P, K, FW, and DM mirror the response curves, with increasing differentiation beginning 15 DAT. The strongest treatment effects can be seen for N, with 95-98% of variance resulting from differences between treatments from 18 DAT onwards. 87-96% of variance in tissue K concentration were also explained by treatment effects for this same period. For P, the dominance of treatment effects does not stabilize above 55% until 23 DAT. Treatment effects are the most stable on the DM, maintaining values between 61-70% throughout the experiment. S, Ca, and Mg tissue concentrations showed the least strong treatment effects, with sulfur only beginning to show treatment effect dominance after 23 DAT.

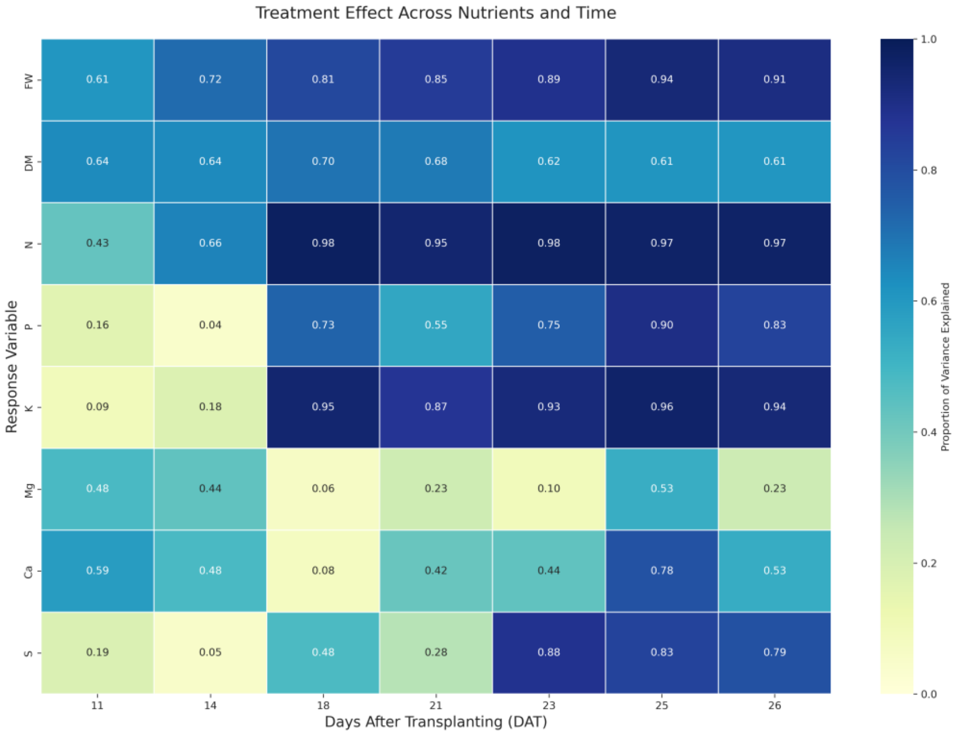

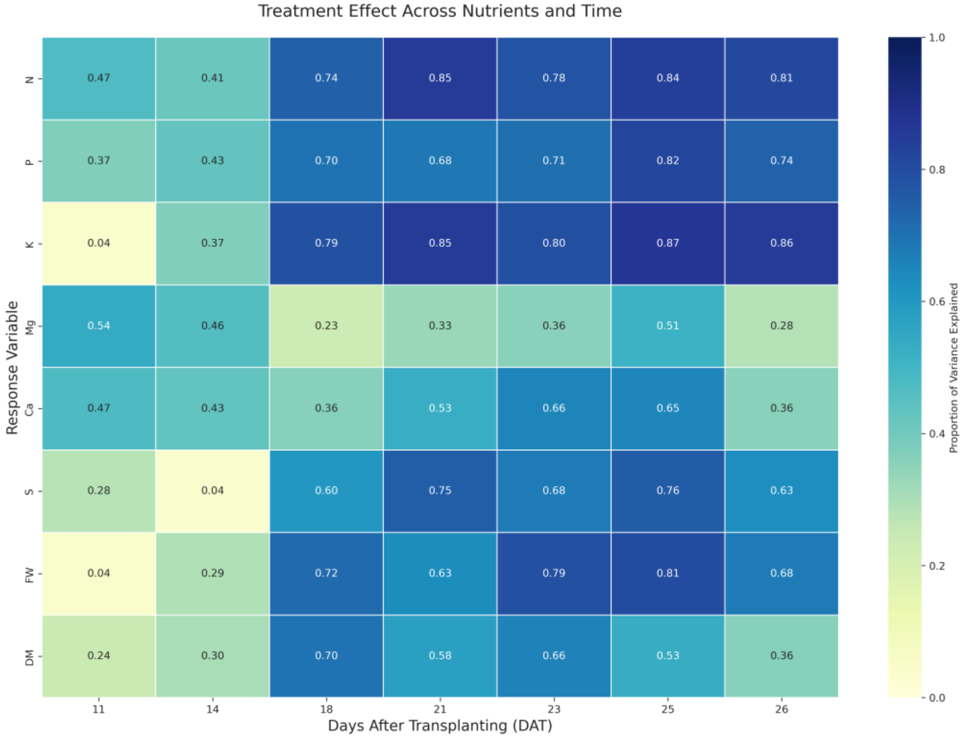

Figure SI 12: Treatment effects across ground truth response variables (top) and RVs estimated by the RF model using daily average VI-based features (bottom).

The most pronounced treatment effects can be seen in estimated N, P, and K trajectories, reaching at least 68% of variance explained by treatment effects by 14 DAT. Mg, Ca, and DM show the lowest treatment

effects, with Mg and Ca never breaking 70% of variance explainable by treatment effects for Mg and Ca, and only reaching 66% at 23 DAT for DM but staying below 65% for the remainder of growth.

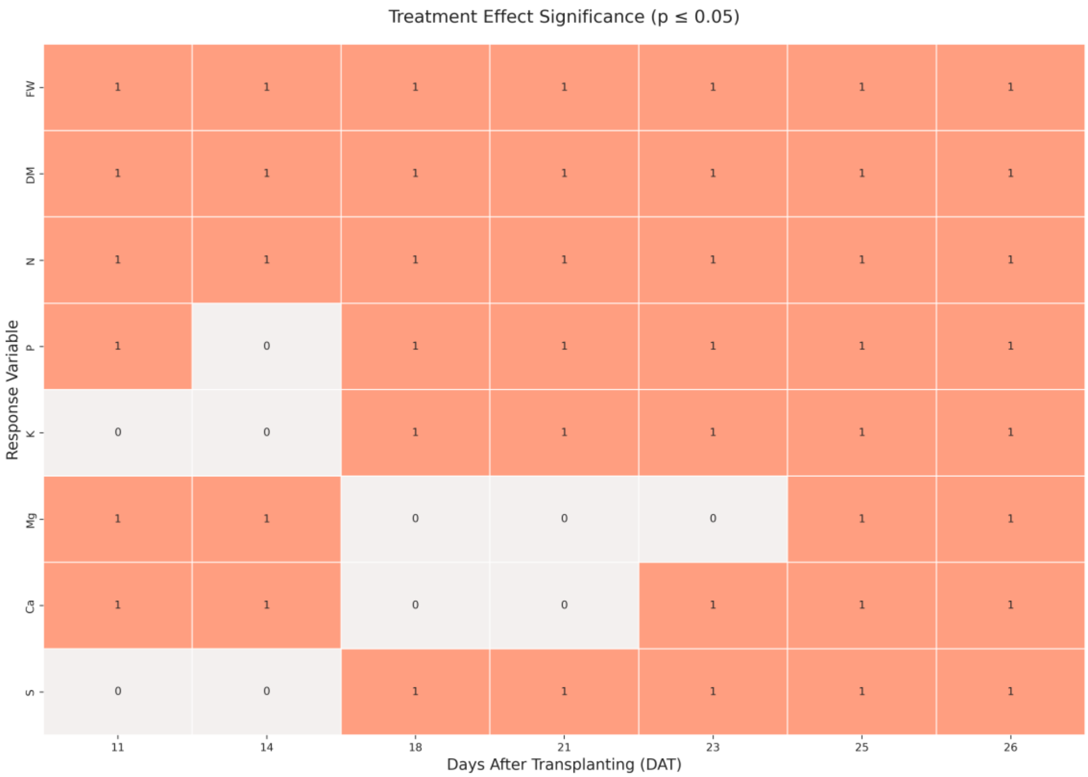

Figure SI 13: Treatment effects significance ($p \leq 0.05$) on GT response variables.

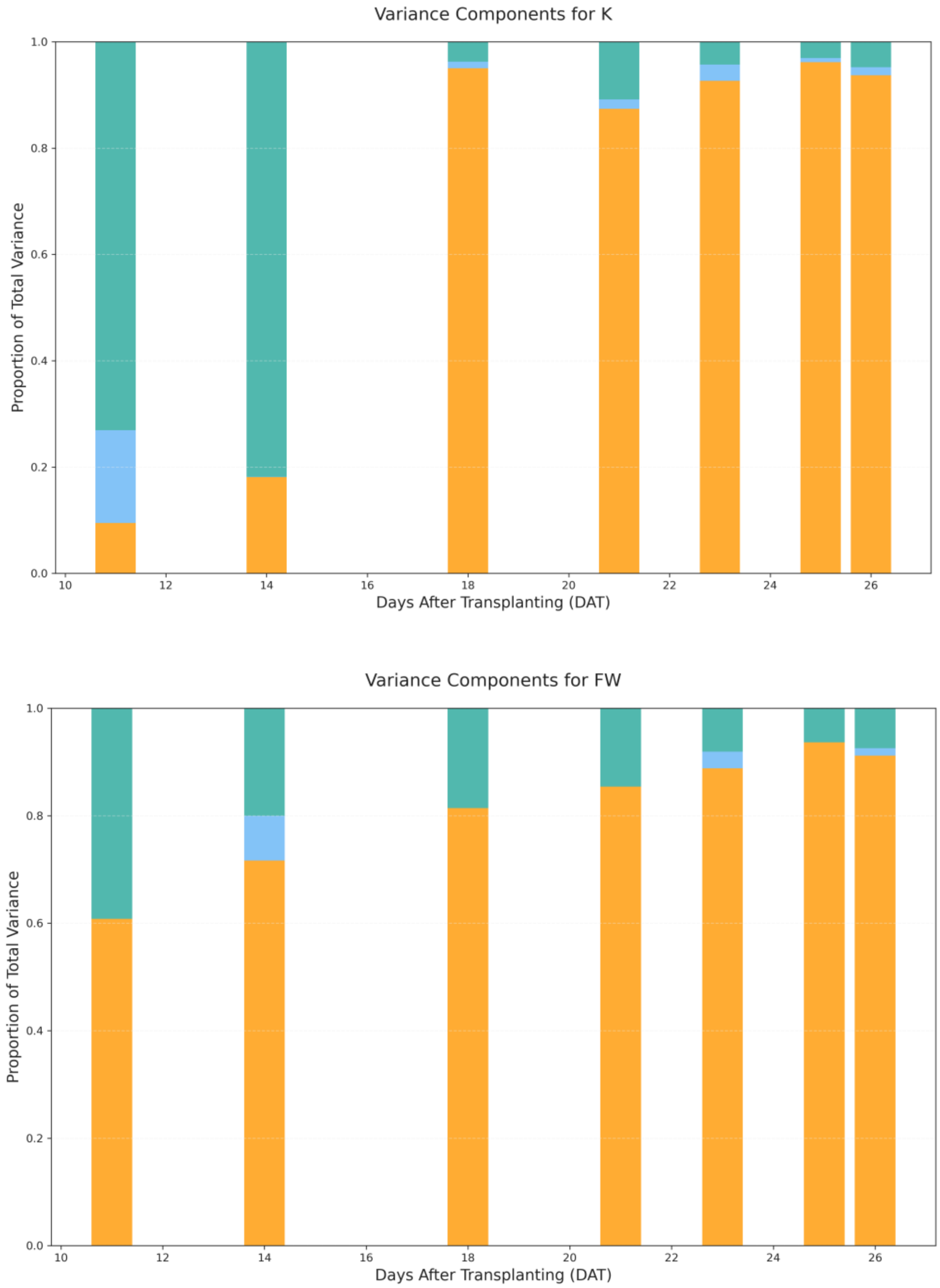

Figure SI 14: Partitioning of total variance between treatment, tank within treatment, and residual (plant, sampling, microenvironment, etc.) for K (top) and FW (bottom).

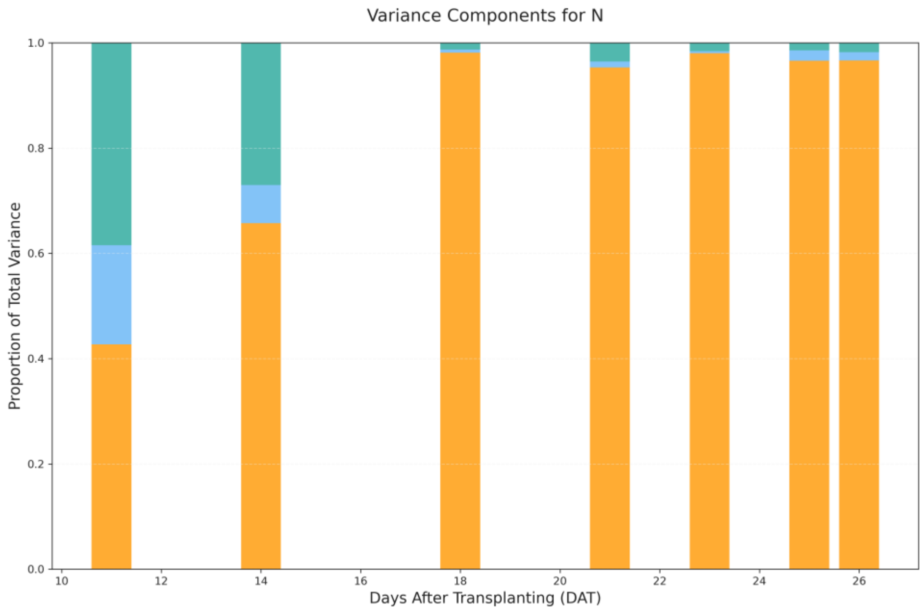

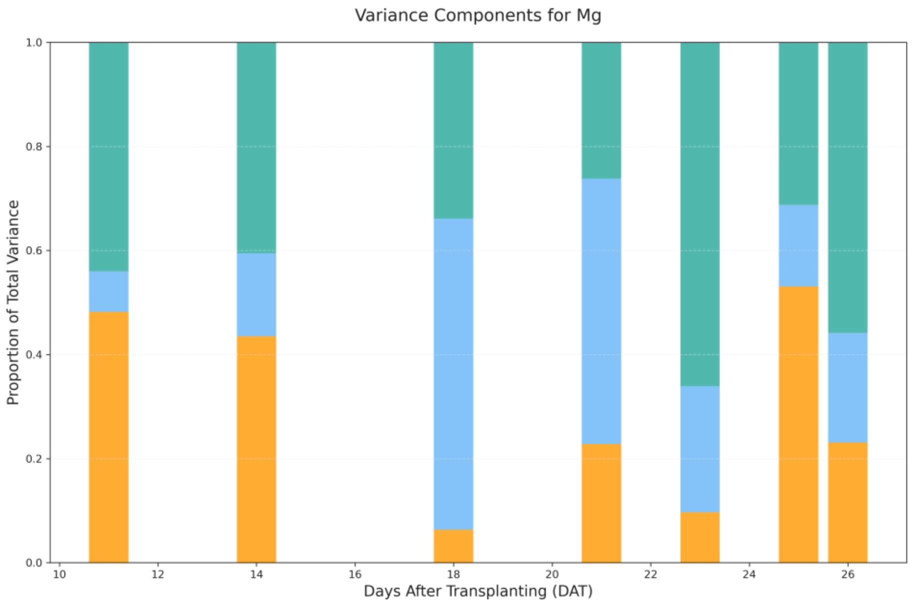

Figure SI 15: Partitioning of total variance between treatment, tank within treatment, and residual (plant, sampling, microenvironment, etc.) for N (top) and Mg (bottom).

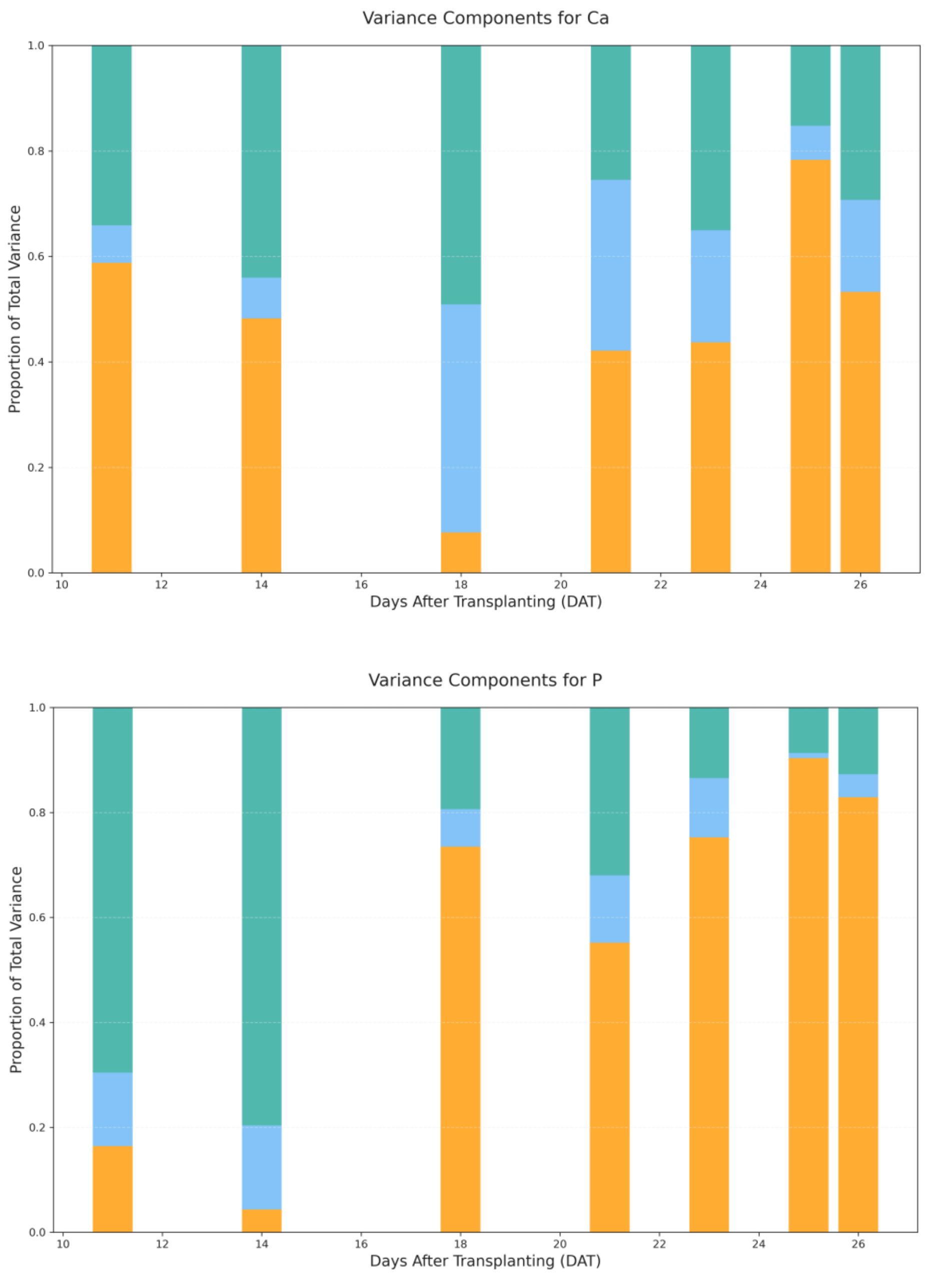

Figure SI 16: Partitioning of total variance between treatment, tank within treatment, and residual (plant, sampling, microenvironment, etc.) for Ca (top) and P (bottom).

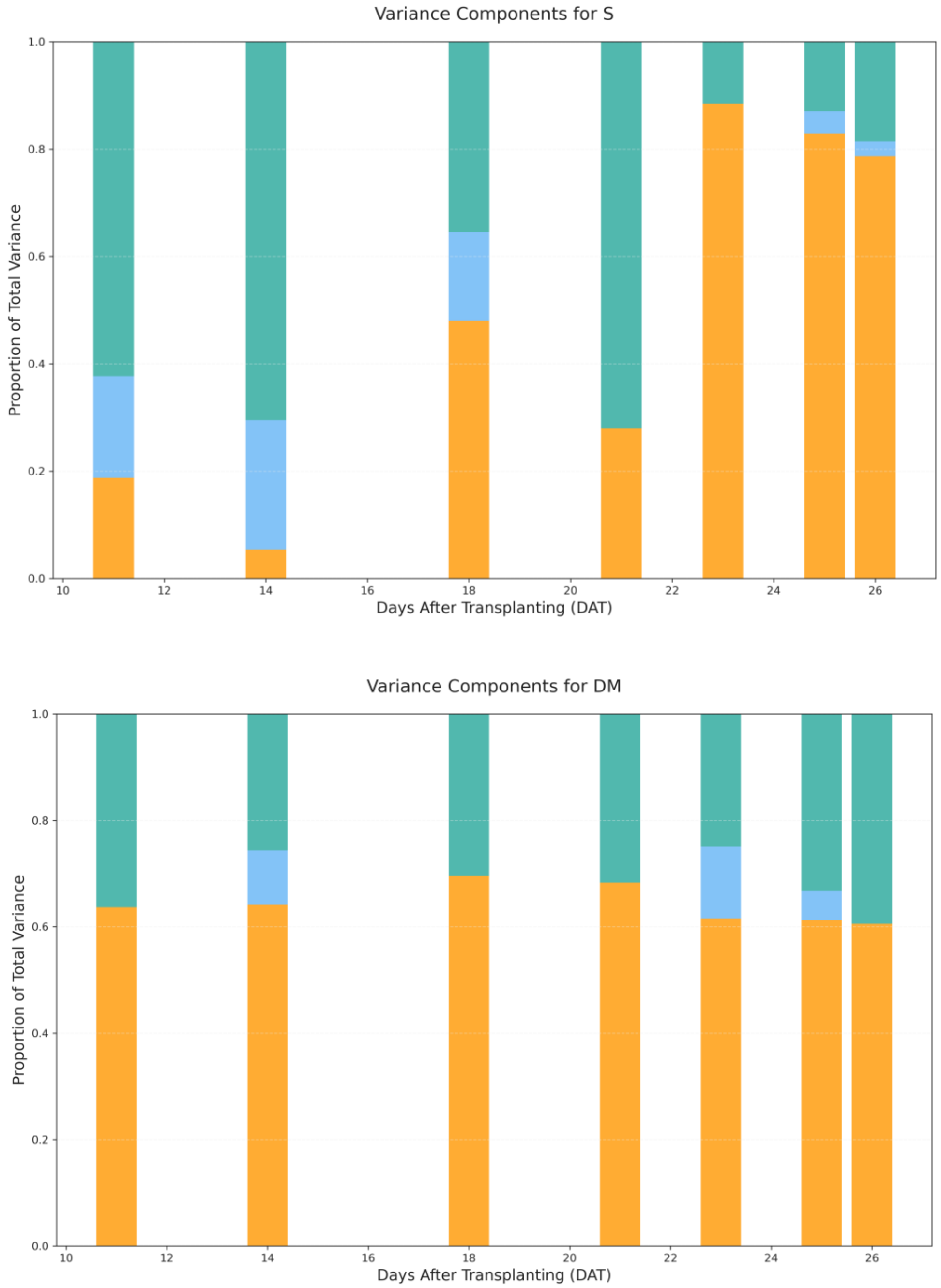

Figure SI 17: Partitioning of total variance between treatment, tank within treatment, and residual (plant, sampling, microenvironment, etc.) for S (top) and DM (bottom).

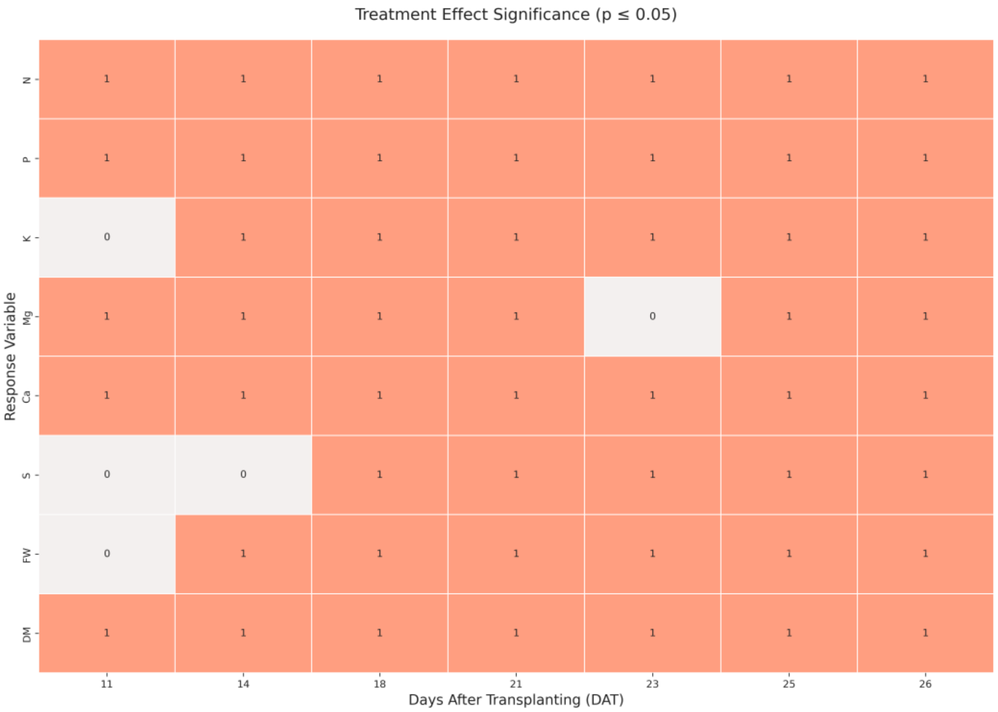

Figure SI 18: Treatment effects significance (p≤0.05) on response variables predicted by RF.

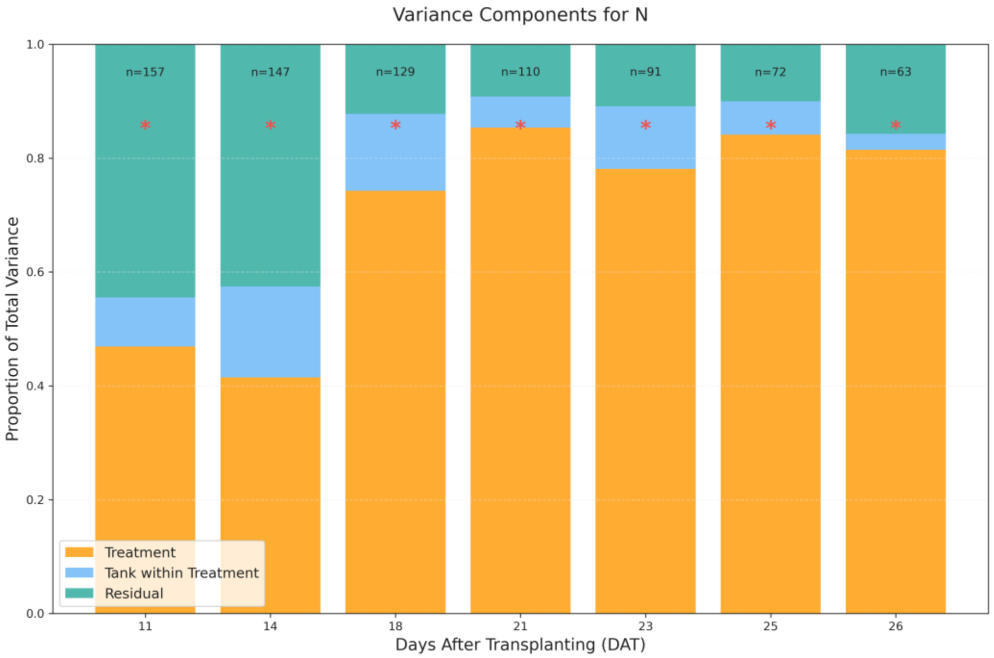

Figure SI 19: Visualization of variance partitioning in the estimated N response using the RF model for each day from 11 DAT.

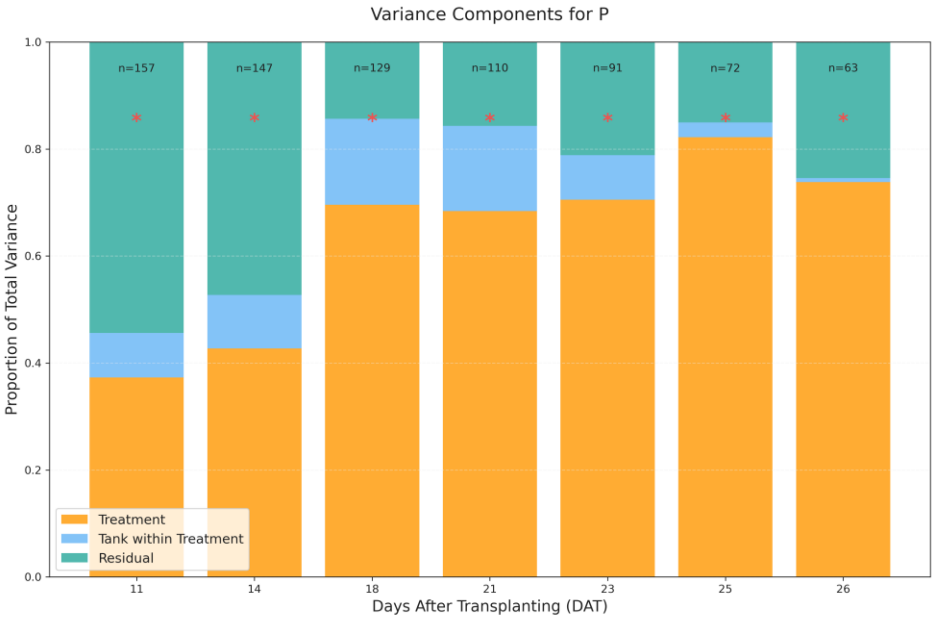

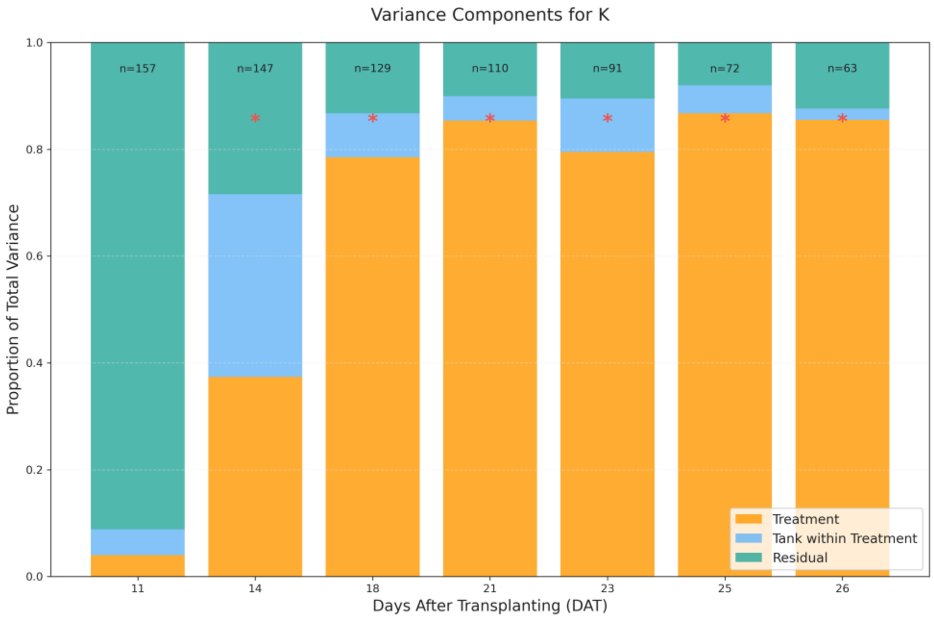

Figure SI 20: Visualization of variance partitioning in the estimated P (top) and K (bottom) response using the RF model for each day from 11 DAT.

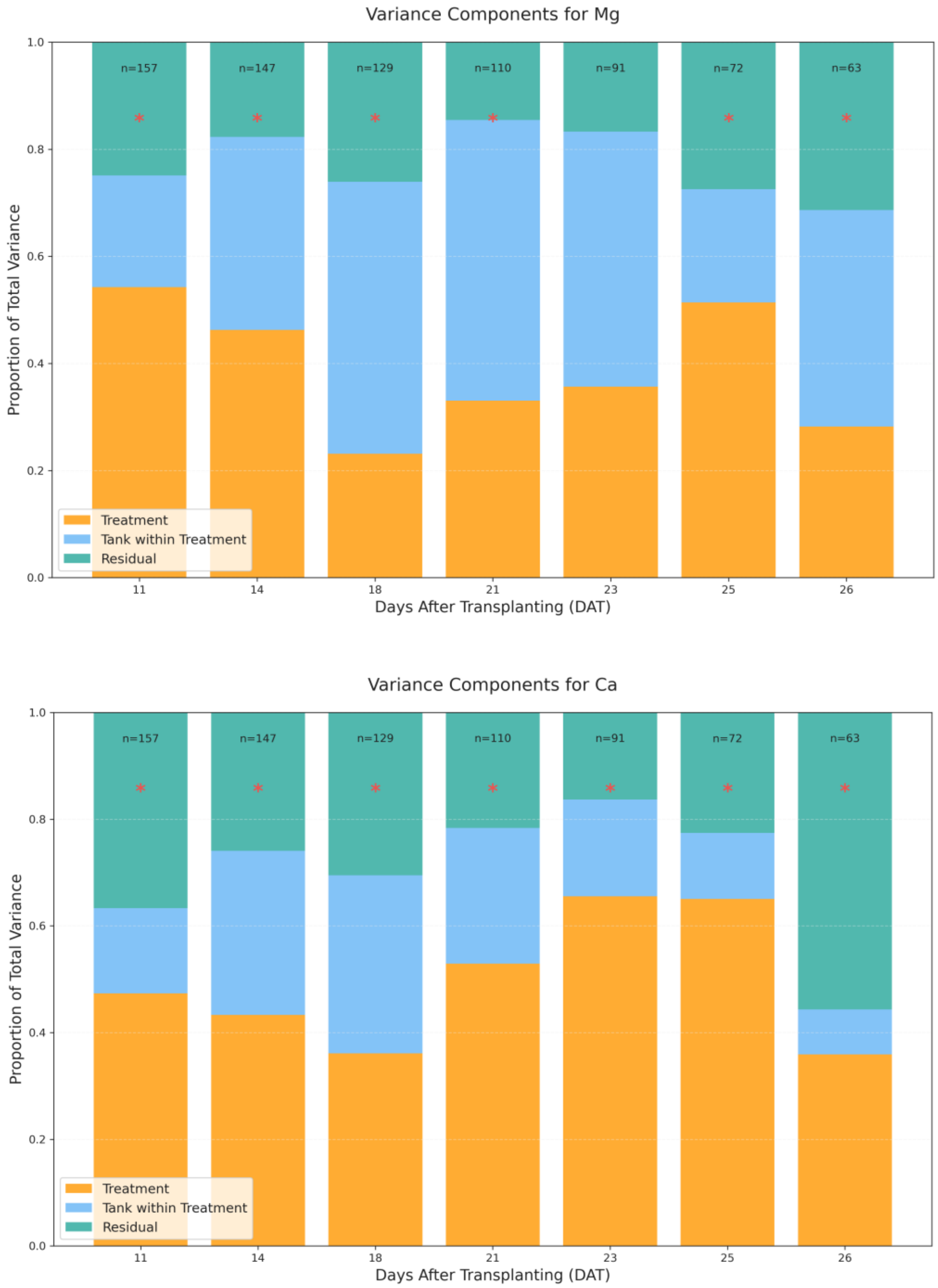

Figure SI 21: Visualization of variance partitioning in the estimated Mg (top) and Ca (bottom) response using the RF model for each day from 11 DAT.

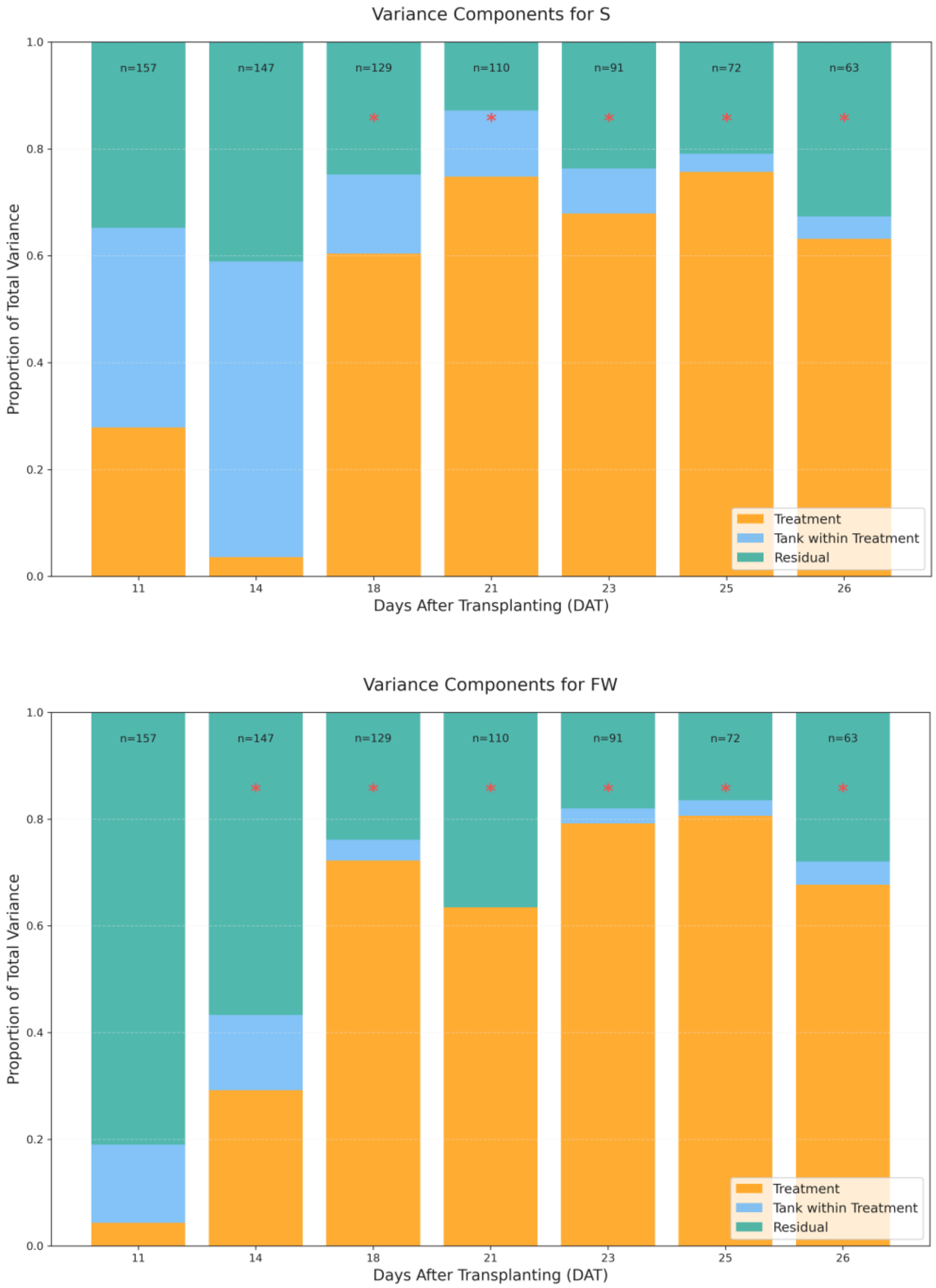

Figure SI 22: Visualization of variance partitioning in the estimated S (top) and FW (bottom) response using the RF model for each day from 11 DAT.

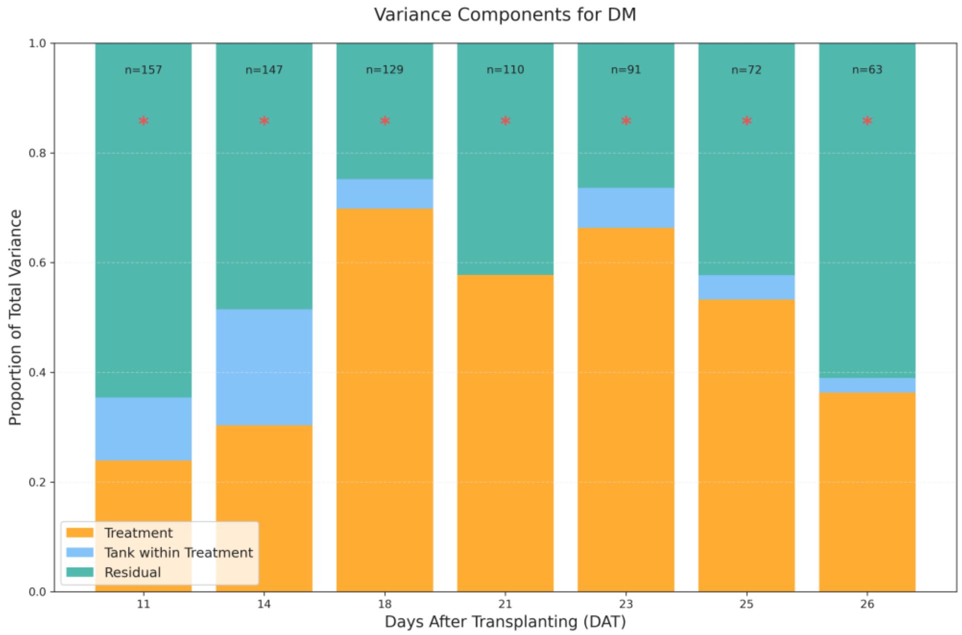

Figure SI 23: Visualization of variance partitioning in the estimated DM response using the RF model for each day from 11 DAT.

REFERENCES

(1) Woebbecke, D. M.; Meyer, G. E.; Von Bargen, K.; Mortensen, D. A. Color Indices for Weed Identification Under Various Soil, Residue, and Lighting Conditions. *Transactions of the ASAE* **1995**, *38* (1), 259–269. https://doi.org/10.13031/2013.27838.

(2) Meyer, G. E.; Neto, J. C. Verification of Color Vegetation Indices for Automated Crop Imaging Applications. *Comput Electron Agric* **2008**, *63* (2), 282–293. https://doi.org/10.1016/J.COMPAG.2008.03.009.

(3) Tucker, C. J. Red and Photographic Infrared Linear Combinations for Monitoring Vegetation. *Remote Sens Environ* **1979**, *8* (2), 127–150. https://doi.org/10.1016/0034-4257(79)90013-0.

(4) Zhang, X.; Zhang, F.; Qi, Y.; Deng, L.; Wang, X.; Yang, S. New Research Methods for Vegetation Information Extraction Based on Visible Light Remote Sensing Images from an Unmanned Aerial Vehicle (UAV). *International Journal of Applied Earth Observation and Geoinformation* **2019**, *78*, 215–226. https://doi.org/10.1016/J.JAG.2019.01.001.

(5) Verrelst, J.; Schaepman, M. E.; Koetz, B.; Kneubühler, M. Angular Sensitivity Analysis of Vegetation Indices Derived from CHRIS/PROBA Data. *Remote Sens Environ* **2008**, *112* (5), 2341–2353. https://doi.org/10.1016/J.RSE.2007.11.001.

(6) Sellaro, R.; Crepy, M.; Trupkin, S. A.; Karayekov, E.; Buchovsky, A. S.; Rossi, C.; Casal, J. J. Cryptochrome as a Sensor of the Blue/Green Ratio of Natural Radiation in Arabidopsis. *Plant Physiol* **2010**, *154* (1), 401–409. https://doi.org/10.1104/PP.110.160820.

(7) Kataoka, T.; Kaneko, T.; Okamoto, H.; Hata, S. Crop Growth Estimation System Using Machine Vision. *IEEE/ASME International Conference on Advanced Intelligent Mechatronics, AIM* **2003**, *2*, 1079–1083. https://doi.org/10.1109/AIM.2003.1225492.

(8) Bendig, J.; Yu, K.; Aasen, H.; Bolten, A.; Bennertz, S.; Broscheit, J.; Gnyp, M. L.; Bareth, G. Combining UAV-Based Plant Height from Crop Surface Models, Visible, and near Infrared Vegetation Indices for Biomass Monitoring in Barley. *International Journal of*

Applied Earth Observation and Geoinformation **2015**, *39*, 79–87. https://doi.org/10.1016/J.JAG.2015.02.012.

(9) Baret, F.; Guyot, G. Potentials and Limits of Vegetation Indices for LAI and APAR Assessment. *Remote Sens Environ* **1991**, *35* (2–3), 161–173. https://doi.org/10.1016/0034-4257(91)90009-U.

(10) Gitelson, A. A.; Kaufman, Y. J.; Merzlyak, M. N. Use of a Green Channel in Remote Sensing of Global Vegetation from EOS-MODIS. *Remote Sens Environ* **1996**, *58* (3), 289–298. https://doi.org/10.1016/S0034-4257(96)00072-7.

(11) Motohka, T.; Nishida, K.; Motohka, T.; Nishida, K. A Simple and Robust Method for Remote Sensing of Phenology Using Green and Red Reflectance: GRVI-Method. *AGUFM* **2009**, *2009*, B43C-0382.

(12) Gao, B. C. NDWI—A Normalized Difference Water Index for Remote Sensing of Vegetation Liquid Water from Space. *Remote Sens Environ* **1996**, *58* (3), 257–266. https://doi.org/10.1016/S0034-4257(96)00067-3.

(13) Fu, Z.; Zhang, J.; Jiang, J.; Zhang, Z.; Cao, Q.; Tian, Y.; Zhu, Y.; Cao, W.; Liu, X. Using the Time Series Nitrogen Diagnosis Curve for Precise Nitrogen Management in Wheat and Rice. *Field Crops Res* **2024**, *307*, 109259. https://doi.org/10.1016/J.FCR.2024.109259.

(14) Cao, Q.; Miao, Y.; Wang, H.; Huang, S.; Cheng, S.; Khosla, R.; Jiang, R. Non-Destructive Estimation of Rice Plant Nitrogen Status with Crop Circle Multispectral Active Canopy Sensor. *Field Crops Res* **2013**, *154*, 133–144. https://doi.org/10.1016/J.FCR.2013.08.005.
